# Fundamentals of Fast Tsunami Wave Parameter Determination Technology for Hazard Mitigation

**DOI:** 10.3390/s22197630

**Published:** 2022-10-08

**Authors:** Mikhail Lavrentiev, Konstantin Lysakov, Andrey Marchuk, Konstantin Oblaukhov

**Affiliations:** Institute of Automation and Electrometry SB RAS, 630090 Novosibirsk, Russia

**Keywords:** tsunami hazard mitigation, early warning technology, fast data processing

## Abstract

This paper describes two basic elements of the smart technology, allowing us to bring to a new level the problem of early warning and mitigation of tsunami hazards for the so-called near zone events (when a destructive tsunami wave reaches the nearest coast in tens of minutes after the earthquake). The sensors system, installed in a reasonable way (to detect a wave as early as possible), is capable of transmitting the necessary raw data (measured wave profile) in a real time mode to a processing center. The smart (based on mathematical theory) algorithm can reconstruct an actual source shape within a few seconds using just a part of the measured wave record. Using modern computer architectures (Graphic Processing Units or Field Programmable Gates Array) allows computing tsunami wave propagation from the source to shoreline in 1–2 min, which is comparable to the performance of a supercomputer. As is observed, the inundation zone could be evaluated reasonably correctly as the coastal area below two thirds of the tsunami wave height at a particular location. In total, the achieved performance of the two above mentioned algorithms makes it possible to evaluate timely the tsunami wave heights along the coastline to approximate the expected inundation zone, and therefore, to suggest (in case of necessity) evacuation measures to save lives.

## 1. Introduction

Seismic events that cause catastrophic coastal flooding due to tsunamis (which have increased in number over the past decade) have a profound impact on the population of such coastal areas. The tragic events of The Great Tohoku Earthquake (on 11 March 2011) have shown that the problem of protecting people and engineering infrastructure from natural seismic disasters is far from being resolved. Tsunami waves generated by this earthquake washed away cars and houses along the northeastern coast of Japan. The Center for Disaster Management and Risk Reduction (Eggenstein-Leopoldshafen, Germany) estimates that about 20,000 people died and more than 1,000,000 lost their homes. More than a million buildings were damaged [1]. 

The tsunami after the Great Tohoku Earthquake refers to a so-called near-field event, where a tsunami wave generated by an undersea earthquake reaches populated areas of the coast very quickly. This time is only about 20 min in the case of the offshore earthquake in Japan. Consequently, all elements of the warning system have to work practically in data ingress mode.

There are several available software packages for tsunami numerical modelling. Among the most known we mention the following ones: MOST (Method of Splitting Tsunamis, NOAA Pacific Marine Environmental Laboratory, Seattle, WA, USA) [2,3]; COMCOT (Cornell University, Ithaca, NY, USA; Institute of Geological & Nuclear Science, Lower Hutt City, New Zeland) [4]; TUNAMI-N1/TUNAMI-N2 (Tohoku University, Sendai, Japan) [5,6]; VOLNA – tsunami code (Centre de Mathmatiques et de Leurs Applications, Cedex, France; School of Mathematical Sciences, Dublin, Ireland) and Numerical tsunami model NAMI-DANCE (Special Bureau of Sakhalin, Russia and METU, Turkey) [7]. In order to obtain results in the shortest time, some algorithms are implemented on GPUs and high-performance clusters. However, none of these packages gives a result in the required timeframe of 1–2 min.

All of these tools contain simulation of the three basic process stages, namely: generation, propagation and inundation of dry land. Without going into details, let us say that first we need to obtain data describing the tsunami source (the initial form of displacement of the water surface), then calculate wave propagation over the water area of interest, and finally, determine the inundation zones. At each of these steps, we inevitably have to make assumptions (to some degree justified), and thus, the final result will be very approximate.

As already noted, the central problem for near-flood events is the limited time for modeling. In this paper we describe an approach based on modern (smart) measurement and data analysis techniques that can significantly reduce the time required to obtain a reasonable estimate of expected wave heights along the shoreline, and thereby approximate the inundation area.

The rest of the paper is arranged as follows: In the Materials and Methods Section we first provide a brief introduction to the existence systems of tsunami wave measurement. Focus is given on bottom-based pressure sensors (floating buoys and sea-bed cables). Then the way to optimize the sensor network is described. Within the “calculation in advance” smart data inversion strategy it is possible to obtain an approximation of tsunami parameters at source using just a part (slightly exceeding a quarter period) of the wave first period. It saves time for the further data analysis. The last part of the Section is devoted to the smart calculation of the wave propagation over the considered water area. By using the FPGA-based Calculator as the co-processor, calculation takes nearly 1 min for a modern personal computer. The achieved performance is comparable to that of a supercomputer. Numerical experiments are described in the corresponding Section. Approximations of the 11 March 2011 tsunami, available in literature, are used. The obtained results are discussed.

## 2. Materials and Methods

The essence of the proposed approach boils down to four elements: (1) modeling of a tsunami wave is based on the displacement of the water surface in the tsunami source, obtained by recalculating the wave profile measurements in those terms; (2) using modern (smart) algorithms to estimate this displacement by a part of the first period of the wave recorded only in one point; (3) calculating the wave on a personal computer with a hardware gas pedal based on FPGA locally for populated areas of the coast, using the nested grid method; and (4) assessing the flooded area based on the estimates of expected wave amplitude.

### 2.1. Data for Analysis

First, it is necessary to obtain input data for wave modeling, namely seafloor deformation parameters, at the source of the tsunami. An internationally developed network of seismic stations gives the coordinates of the earthquake epicenter and its magnitude (an estimate of the energy released) virtually immediately after the earthquake. An earthquake with a magnitude greater than M > 7.5 usually generates a dangerous tsunami. Most modern tsunami warning systems convert this information into an assumption about the shape of the seafloor deformation at the tsunami source. They use information on the geological structure of the focal zone, numerical calculations of residual displacements in an elastic half-space [8,9], and analysis of historical data on seismic events in the area, etc.

Since the result is still only an approximation, we consider it promising to take ocean level measurements as the basis for the propagation of an already formed wave. Several approaches are currently used to solve this problem. Without going into an analysis of the strengths and weaknesses of the various methods, we will rely on direct measurements of the parameters of the already generated tsunami wave on the water surface. We will limit ourselves to a brief listing of the methods used; a more detailed analysis can be found in [10]. Among the sources of information used we note: tidal gauges, ultrasonic and pressure sensors, GPS sensors, and space altimetry images.

Consider several technologies to obtain sea level measurements. Tide gauges are abundant, but when installed on the shoreline, they provide data that are extremely noisy due to multiple reflections from the shoreline that are distorted by bottom friction and wave toppling. In addition, these data are completely unsuitable for tsunami warning because they provide data after the wave arrives on shore. Usually, they are used for post-event analysis and reconstruction of the actual tsunami source. 

Methods based on the analysis of data from GPS sensors installed on land have worked well in Indonesia, for example. However, at this time it is not certain that this technology will work as well in other regions. Sea-based sensors—the so-called GPS buoys—are currently technologically possible to install only in shallow depths (300, and in the future, up to 600 m), which imposes a severe limitation on the advance of wave data acquisition. Such buoys installed to the east off the Japanese coast successfully detected the far-field tsunami generated by the 2010 Chile Earthquake [11].

Altimetry data from low-flying satellites are highly accurate and could form the basis of a warning system. However, these satellites are not geostationary and are unlikely to be in the right place at the time of a seismic event.

The most reliable and promising at present, in the opinion of the authors, are bottom-installed pressure sensors that allow measuring the height of the tsunami wave with centimeter accuracy, and transmitting these data to a processing center via satellite communication channels [12]. Until the beginning of this century, there were no technical means of recording a moving tsunami wave in the deep ocean (i.e., at depths greater than 100–200 m) that would carry information about the tsunami source free of distortion by friction on the bottom and multiple reflections from the shoreline. Therefore, attempts have been made to estimate expected wave heights near the shore from seismic records. However, no clear dependence of the height of the initial vertical displacement in the tsunami source on the earthquake magnitude, which seismologists are able to determine quickly enough (usually a few minutes after the seismic event), has yet been found. Therefore, apart from making a statement about the occurrence of a tsunami, no quantitative estimates of wave height can yet be made using only seismic data. With the advent of deep ocean tsunami recorders integrated into the DARTS (Deep-ocean Assessment and Reporting of Tsunamis) system, it has become possible to measure actual tsunami heights in some points of the Pacific and other oceans [12].

These sensors are located in the deep ocean opposite the subduction zones [13], in which almost all epicenters of tsunamigenic earthquakes are located. Since the speed of wave propagation (estimated within the well-proven shallow water theory approximation) is proportional to the square root of the depth, the wave reaches at least one of these sensors much faster than it reaches the shore.

**Figure 1:**
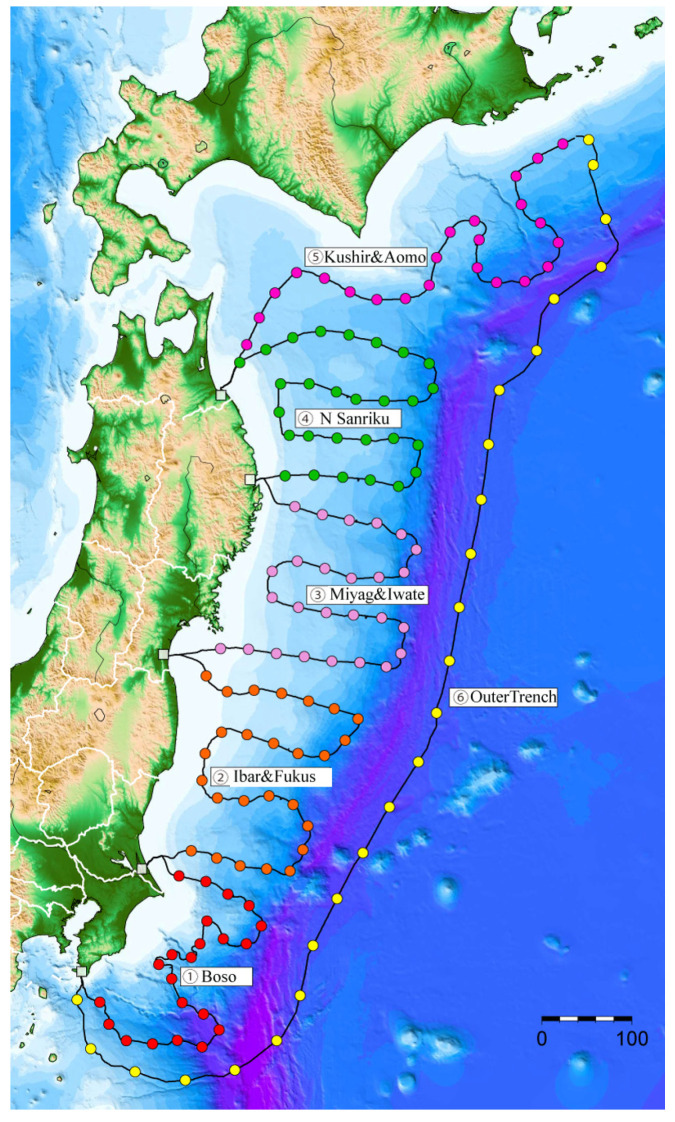
Configuration of S-net cable system off the northeast Japan coast for seismic and tsunami wave detection [15]. Numbers indicate related areas: 1—Boso, 2—Ibaraki & Fukushima, 3—Miyagi & Iwate, 4—Northern Sanriku, 5—Kushiro & Aomori, 6—Outer trench.

The approach implemented in Japan should also be noted. To determine the vertical displacement of the ocean surface around and inside the tsunami source, two zones of underwater earthquake epicenters were covered by a network of sensors connected by a bottom cable. Of the rather broad functionality of the sensors, we are interested in the pressure sensors. One such network, NIED S-net (Sea-floor observation network for earthquakes and tsunamis along the Japan Trench) [14], consists of 150 observation units from off Hokkaido to off Chiba Prefecture. Configuration of this network (taken from [14]) is presented in Figure 1. Each unit contains seismometers and water pressure gauges to monitor offshore earthquakes and tsunamis. All the data are transmitted to the land stations by fiber-optic cable and arrives at NIED in real time [15,16]. 

The second system, developed in Japan, covers a part of Nankai trough and is called DONET (Dense Oceanfloor Network System for Earthquakes and Tsunamis). The first phase of this program has been carried out since 2006 with the purpose of monitoring the earthquake epicenter’s region close to Nankai trough, and the installation of observational equipment on 20 stations at Kumanonada was completed in 2011. The second phase (DONET2) has also started to cover a wider region in 2010. A total of 29 observatories are planned to be installed at offshore Kii peninsula for DONET2 and 2 additionally at Kumanonada for DONET [17]. These observation networks successfully detected a few events. For example, S-net stations recorded the 2016 Fukushima tsunami, whereas DONET stations recorded the 2022 Tonga volcanic tsunami [18].

To conclude, we state that direct measurements of tsunami wave at deep ocean are available for analysis in a real time mode.

### 2.2. Smart Sensor Network Design to Identify Source Parameters 

Let us consider the possibility of determining the approximate parameters of the wave in the tsunami source in the shortest possible time. We will take as a basis the direct measurements of the wave profile by the bottom pressure sensors. Except for the already mentioned S-net and DONET networks, the existing sensors (DART buoys of USA NOAA and similar ones produced in other countries) are located in front of the selected subduction zones quite randomly, not accounting for wave travelling times and over parameters that are critical for timely warning about tsunami wave danger. 

At the same time, mathematical modeling methods can easily solve the problem of optimizing the locations of a small number of additional (to the already existing ones) sensors in order for the tsunami wave to reach the nearest sensor in the minimum possible time [19]. It should be noted that this will be the guaranteed time in the so-called worst-case scenario, when the epicenter of the earthquake within a given subduction zone will be as far (in terms of wave propagation time) from the sensor system as possible. Until now, this approach of “smart” [20,21] extension of the observation system has unfortunately not been developed.

In a few words, the essence of the method of reconstruction of the initial displacement in the source using data from several deep-water recorders is as follows. The sensor wave record is a sequence of ocean level measurements *f(n −* Δ*t), n* = 1, ..., *N* with fixed time step Δ*t*. To reconstruct the source, we first numerically simulate tsunami propagation from each UnS_k_, *k =* 1, ..., *K* and store the wave signals (synthetic mareograms) *f_k_(n −* Δ*t), n* = 1, ..., *N* at sensor locations with the same time step as in the real recordings. The recovery process consists of finding the set of coefficients *b_k_*, which provide minimum of discrepancy between the real recorded signal and the linear sum of synthetic mareograms from several UnSs according to the following discrete version of mean square difference:

(1) $${\sum_{n=1}^{N}\left(\sum_{k=1}^{K}b_{k}f_{k}(n\cdot \Delta t)-f(n\cdot \Delta t)\right)}^{2}\Delta t\to \min$$

The result will approximate the water surface in the source by linear combination of displacements in UnSs with found coefficients *b_k_*. The procedure of orthogonalization and normalization of discrete synthetic mareograms (in fact, vectors) allows us to find the required set of coefficients in a few seconds, regardless of the number of involved UnSs. Detailed formulae (computationally low cost) for finding the desired coefficients *b_k_* could be found in [21]. 

Preliminary numerical calculations for model sources show that optimizing the location of the sensors opposite the 1000 km long subduction zone makes it possible to register a wave no later than 10 min after the earthquake with only 4 DART buoys. It is believed that only the whole period of the wave carries sufficient information to make a correct judgment about the source parameters. Moreover, since the form of the initial disturbance of the water surface we are interested in is two-dimensional, and the record of the profile of the wave passing over the sensor has only one dimension, from the mathematical point of view several (according to different estimates from 3 to 10) records from different sensors are required as input data. In the works, where such recovery is carried out from the data of 3 sensors [22,23], their “ideal” location in relation to the source is required. Thus, for the practical application of this approach, several dozens of sensors must be installed in front of a 1000 km long subduction zone.

In fact, knowledge of the detailed structure of the initial waveform is not necessary to assess the tsunami hazard. The most important parameters are the size of the disturbance zone and the amplitude of the wave at the initial time instance. Model calculations [24] show that this information can be obtained within the concept of pre-computations using about one quarter of the first wave period (if the deep-water recorder is located on the side of the positive wing of the dipole source), recorded at only one point. Already in the case of 50 km wavelength (which corresponds to 250 s of its period passing over the sensor), the result of amplitude determination is obtained by 3 min before the wave passes over the sensor completely. This time gain becomes even more significant for longer waves, typical for catastrophic events. The algorithm itself, based on Fourier series theory, has low computational complexity [20,21]. The recovery time takes a few seconds when using a personal computer (PC).

For tsunami warning services, the location of the observing system should be of interest so that after determining the parameters (amplitude) of the tsunami source, there is as much time as possible before the wave arrives on shore. Model calculations show that in this case the optimal location of the sensors should be different from the one that provides the shortest time to determine the parameters [21,24]. Until now, the authors are not aware of the practical application of this technology.

**Figure 2:**
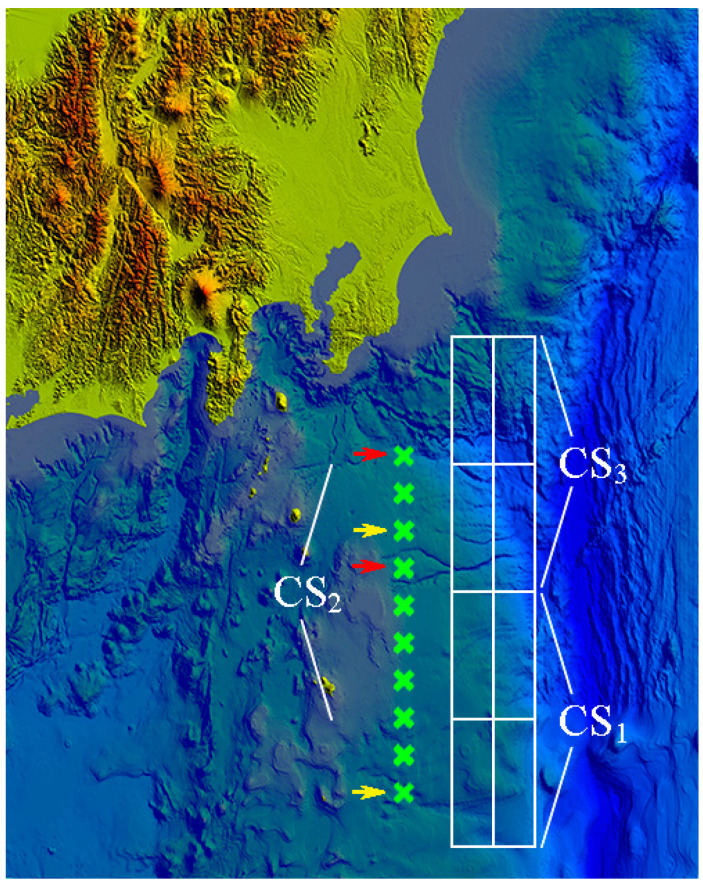
Digital bathymetry used for numerical tests. Positions of artificial combined tsunami sources *CSi* are indicated. Model sensors are located at green crosses. Red and yellow arrows show optimal positions of sensors depending on the criteria considered.

Figure 2 shows a digital grid bathymetry off the southeastern coast of Japan (Honshu Island), constructed based on data from the Japanese Agency JODC [25]. The white rectangles show the location of tsunami “unit sources” (UnSs) with the shape characteristic typical for this seismic area. In the calculations, we used “Composite sources” *CSi,* which are the initial surface displacement obtained as the sum of four neighboring UnSs with some coefficients. The form of such a composite source *CS* well simulates real historical events. Green crosses denote the positions of model sensors *L*1*–L*10, the first of which *L*1 is located on top, *L*2 below it, and so on down to the lowest *L*10.

The times in which the proposed algorithm determines source parameters of *CSi* (*i* = 1, 2, 3) if data from each of the sensors *Lj* (*j* = 1, ..., 10) were used have been obtained by numerical tests. In addition, for each sensor, the time it takes for the tsunami wave to reach the nearest point on the coast after the source parameters has been determined. Data on the optimal location of the sensors are shown in Table 1.

The optimal location of the two sensors in terms of the shortest time to determine the source parameters (for the considered three positions *CSi*, *i* = 1, 2, 3) is shown in Figure 2 by yellow arrows. The location of the two sensors in the places marked by red arrows gives the maximum time between the source parameters determining and wave arrival to the nearest shore, Table 2.

Thus, the correct (smart) location of the sensor network and the algorithm of data circulation based on mathematical theory can allow us to obtain reasonable approximate values of parameters of the initial displacement of the water surface in the tsunami source in a comparatively short time. 

### 2.3. Smart Data Processing: Fast Numerical Modeling

After obtaining an estimate of the initial displacement of the water surface in the source, the stage of numerically solving the Cauchy problem for the system of shallow water equations [26] is followed; this determines the expected distribution of maximum wave heights along the shoreline. Note that this model is widely used for modeling tsunami wave propagation [3,27]. As a practice shows, the distribution of maximum wave heights along the shoreline is highly irregular and characterized by the presence of highly localized peaks. In other words, wave amplitudes can differ many times in neighboring segments of the coast, making selected sites on the coast extremely dangerous. It is known from the theory and practice of difference methods that the results of numerical calculations of problems for partial differential equations can be trusted only if the time step is in a certain dependence on the step length of the spatial grid. This is a consequence of the Lax theorem [28], which causes the convergence of the numerical solution to the exact solution by the requirement of stability and approximation of the original differential equations by the difference scheme. In addition, the stability condition establishes the correlation between the time step value and the spatial step length of the computational grid. For explicit difference schemes the calculation of wave propagation (or other disturbances) is stable if the Courant condition, which limits the wave advance for one time step to one step of the computational grid, is satisfied. It follows that reducing the spatial step requires a smaller time step. Thus, a calculation with a sufficiently detailed spatial computational grid can take too much time, even with a powerful computer or supercomputer. In order to determine the amplitude of the wave approaching the shore, it is necessary to perform calculations on sufficiently detailed grids with a step of about 10 m.

Therefore, solving this standard problem (numerical solution of the shallow water system) requires a significant amount of time, even with modern supercomputers. Note that as the events of 11 March 2011 showed, in the case of a strong earthquake power outages are to be expected, and these may hinder the use of supercomputer systems.

It is possible to combine such mutually exclusive requirements as: (1) grid step at near shore zone is ≤10 m; (2) total time of computation is about 1 min; (3) obtaining results in case of power outage, in the way of application of smart processing algorithms, namely, application of hardware acceleration of calculations, made on a modern personal computer. Thus, the first requirement is fulfilled by using nested grids [29,30]. “Energy-independence” is achieved by using a PC with an uninterruptible power supply. Finally, the required calculation speed is provided by a specialized FPGA-based calculator [31].

The use of FPGA as the hardware basis of computing acceleration has its own limitations. Consequently, the required performance is achieved by programming parallel computational pipelines, which allows start calculations of variable values at the (*n* + 1)-step in time as soon as their values in neighboring grid points at the *n*-th time step become known [32]. In this case, it is required to store all necessary data in the internal memory of each special processor. In a considered case of shallow water equation system these are two components of velocity vector *(u(x,y,t),v(x,y,t))* and total height of liquid column *H(x,y,t) = h(x,y,t) + D(x,y)*, where *h(x,y,t)* stands for the tsunami wave height of interest, and *D(x,y)* is a readily known depth in all points of the area. When using the relatively low-cost solutions VC709 (host computer is required) or ZCU106 (stand-alone solution), there is a limit to the computational grid size of 3000 × 2500 nodes.

Such a restriction allows us to consider a computational area of the order of 1000 × 700 km, which is usually sufficient for near-shore events. Then it is enough to calculate on a sequence of three refining grids, reducing the step from the initial 250–300 m to 10–15 m in the coastal zone. The disadvantage is that the last calculation stage covers a coastal area of about 30 km. This limitation can be compensated by simultaneous calculations on several nested grids of fine resolution [29], or by delegating the calculations to local emergency warning services that are interested in a similarly sized coastal area. Let us reflect that only a PC resource is required for the calculation.

## 3. Numerical Results

Two versions of the source parameters of the catastrophic tsunami (of 11 March 2011) at offshore Japan (The Great Tohoku Earthquake), suggested in the literature, were used in numerical experiments [33,34]. Our goal is to reduce the time required for numerical simulation of tsunami wave propagation by using the earlier proposed hardware-software solution based on Field Programmable Gates Arrays (FPGAs). The FPGA based co-processor in the form of a printed circuit board, called Calculator, can operate as part of a modern PC.

### Numerical Experiments

**Figure 3:**
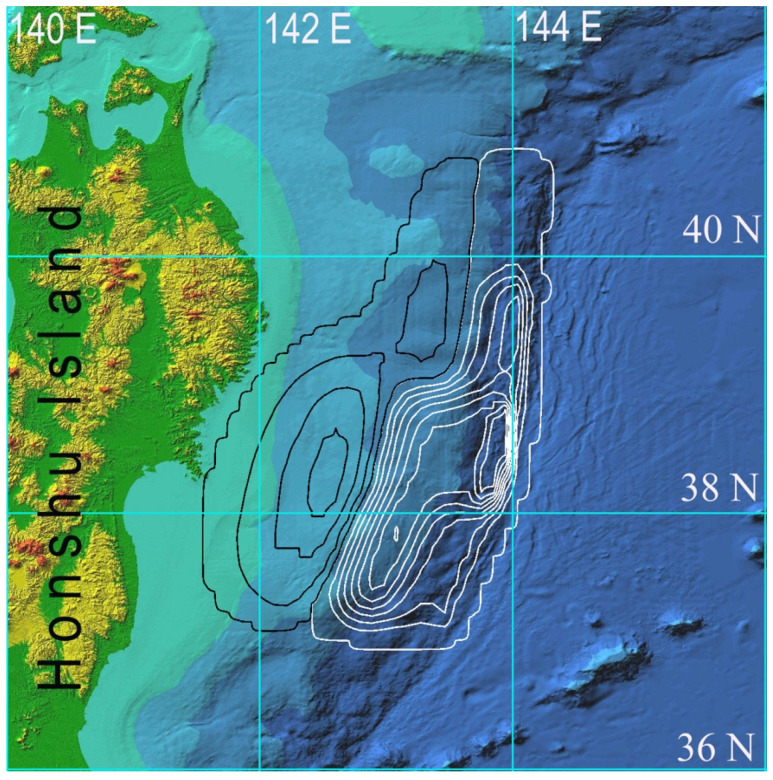
Digital bathymetry and tsunami source of 11 March 2011 event. The isolines of the field of water surface vertical elevation with an interval of 1 m are drawn in white, and the isolines of the initial decrease in water level are drawn in black. The source parts are outlined by ±0.05 m level lines. The maximum elevation in source is limited by +9 m and the lowest value is equal to −4 m.

To demonstrate the performance of the proposed approach to coastal tsunami hazard assessment, numerical calculations of tsunami wave propagation (initiated by the Great Tohoku Earthquake of 11 March 2011) were carried out. The area around the northeastern coast of Honshu Island (Japan), bounded by latitudes 36° N, 42° N and longitudes 140° and 146° E, was selected for numerical modeling. A digital bathymetry array of 2401 × 2401, with spatial steps in both directions equal to 0.00248 arc degrees (214 m in the west–east direction and 276 m in the south–north direction), was prepared based on the JODC bathymetric data [25]. The bottom relief with reference to geographic coordinates is shown in Figure 3.

**Figure 4:**
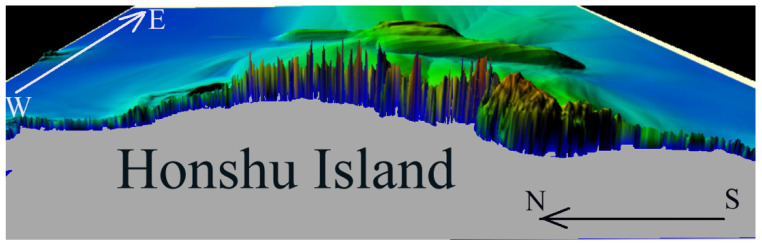
3D visualization of the distribution of tsunami height maxima along the coast of Honshu Island after 6000-s calculation of wave propagation.

The first computational experiment simulated the propagation of the tsunami wave of 11 March 2011, generated by the initial displacement of the water surface (Figure 3) coinciding with the displacement of the ocean floor given in [33]. During the numerical calculation, the maximum water surface level was fixed at each node of the computational grid for the entire simulation time. A 3D visualization of the tsunami height maximum distribution over the entire computational domain is shown in Figure 4.

Figure 4 clearly shows the distribution of maximum wave heights (up to 22.4 m) along the coast. The sharp fluctuations between the maximums along the coastline are explained by the presence of numerous harbors and capes at Sanriku coast. Numerical calculations using the described method did not simulate the process of wave runup on the dry shore. The condition of total wave reflection was implemented along the entire coastline at a depth of 5–10 m, and the data shown in Figure 4 indicate maximum tsunami heights, including those in the nodes of the computational grid closest to the shore. There are several points near the shoreline where the simulation results show tsunami heights exceeding 20 m. In general, the distribution of tsunami heights along the Sanriku coast is like the modeling results described in [33].

**Figure 5:**
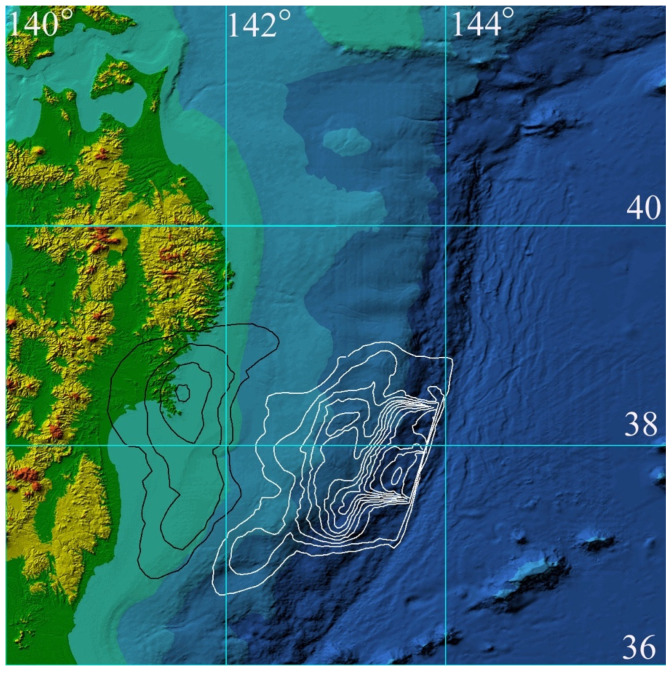
Relief of the seafloor and isolines of the field of initial vertical displacement of the water surface in the source [34]. Isolines of subsidence (colored in black) are represented with an interval of 0.5 m in the range from −0.5 m to −2.0 m, and isolines of level elevation (colored in white) are represented with an interval of 1 m in the range from +1 m to +11 m.

In the second computational experiment in the same grid area of 2401 × 2401 computational nodes as the source of the 11 March 2011 tsunami, the initial water surface (same as bottom) displacement from the publication [34] was used. Its location in the form of isolines of the field of vertical displacements of the water surface is shown in Figure 5.

**Figure 6:**
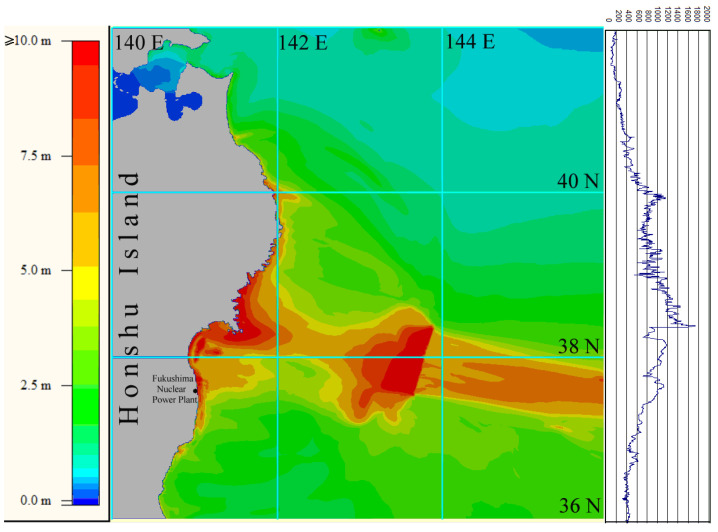
Distribution of tsunami height maxima in the entire computational domain based on the results of numerical tsunami simulation of 11 March 2011 using the source model proposed in [34]. Wave heights distribution (in cm) along the coastline is shown in the right part of the figure.

The numerical calculation proceeded with 12,000 time steps, which correspond to 6000 s of tsunami propagation. Based on the simulation results, the maximum wave heights (during the whole propagation process) were calculated at all nodes of the calculation area. A two-dimensional map of this distribution is shown in Figure 6.

**Figure 7:**
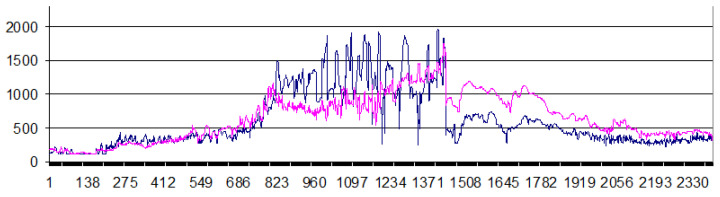
Comparison of maximum tsunami heights (in cm) along the northeastern coast of Honshu Island, obtained using the source model from [33] (blue line) and [34] (pink line).

The color legend in Figure 6 is limited to a wave height of 10 m, although according to simulation results tsunami heights from the source proposed by Fujii in [34] reached 15 m at some points. At the same time, in the Fukushima Nuclear Power Plant area, the maximum tsunami heights according to the calculation results reached 9–9.5 m. Comparison of the distributions of maximum tsunami heights along the coast for different source models is given in Figure 7. The blue line corresponds to the source reconstruction, proposed in [33], while the pink line is based on the source from [34]. The horizontal axis shows the nodes indices along the ordinate axis starting from the northern boundary of the computational domain. 

**Figure 8:**
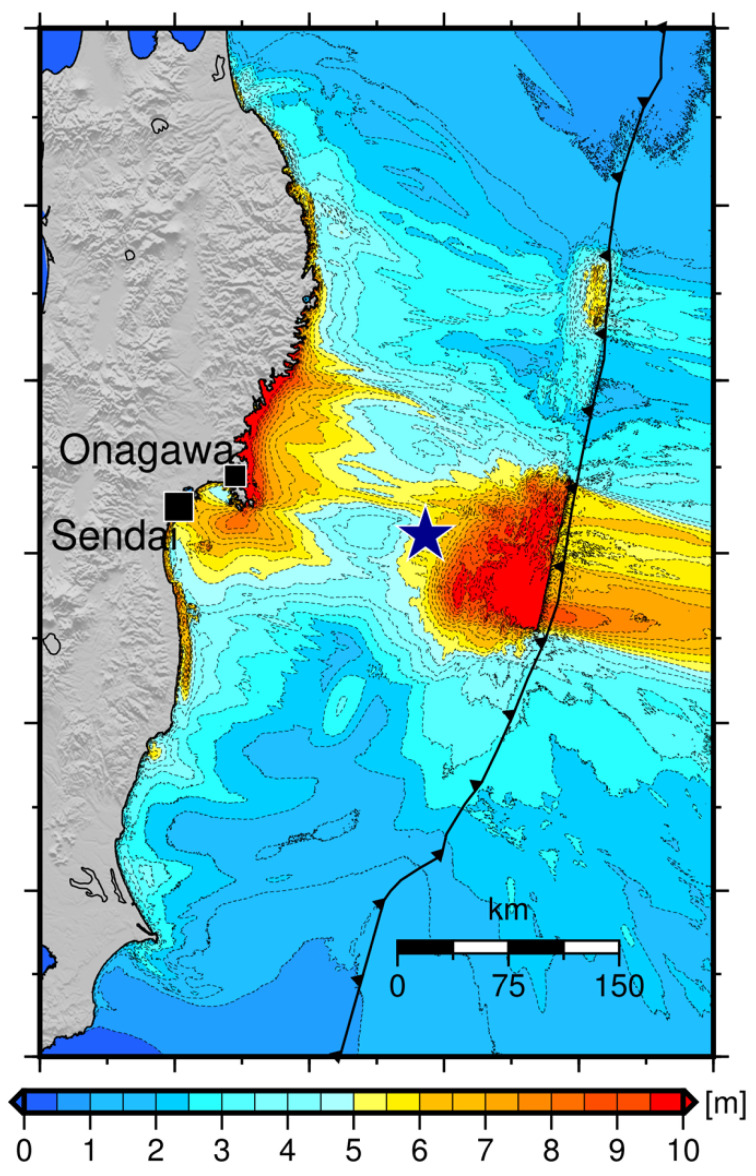
Distribution of tsunami height maxima as a result of numerical calculations performed by Adriano et al. [35] using the source presented in [34].

The distribution of tsunami height maxima, obtained as a result of numerical simulation in the entire computational domain, is generally matching the results of calculations of tsunami propagation carried out by Adriano et al. [35], using the tsunami source of 11 March 2011 built by Fujii [34]. The results of this simulation are shown in Figure 8 as isolines of the distribution of maximum tsunami heights.

## 4. Discussion

Let us summarize how “smart” technologies of sensor location and data processing can contribute to improving the quality of tsunami wave height predictions along the coast. Let us note that the main condition is the running time of the algorithms, which in total should not exceed 1–2 min. 

Firstly, it is required to obtain, as soon as possible, the data of the wave profile measurements. At present, there is a developed system of pressure sensors located in the areas of subduction zones that “surround” the Pacific Ocean water area. These sensors continuously transmit information about the tsunami wave passing over them (more precisely, the height of the water column at a given time) with centimeter accuracy in real time. For this purpose, either satellite communication channels or cables laid on the ocean floor are used [12,13,14,15,16,17]. 

The longest time in this approach is required to wait for the tsunami to reach one or more sensors and record a sufficient part of the wave period for effective source parameters recovery. The procedure itself takes a few tens of seconds to determine the coefficients of the composed source and another 1–2 min to solve the direct problem of tsunami propagation from this source.

To obtain ocean level data in the shortest time the existing observation system can be supplemented “smartly”; that is, in such a way that the optimum value of one or several parameters is obtained [19,24]. Such parameters may be: the minimum time for a wave to reach one of the sensors (when the epicenter of the supposed earthquake passes through the whole considered zone of subduction); the maximum time left for making decisions about evacuation after determining the source parameters; or a combination of these parameters, etc. The cost of installing and maintaining the sensor system can also be included in the criteria system. It is also possible to optimize the surveillance system so that the value of one of these parameters does not exceed some value in the case of failure of one of the sensors. 

The application of the well-developed mathematical Fourier series theory makes it possible, within the framework of the “calculation in advance” concept [3,21], to carry out “smart” processing of the observation system data and obtain approximate values of the source parameters using only part of the first wave period (such part slightly exceed a quarter of entire period). Depending on the wave length, this will save from 1 min (for wave length 20 km) to 5 min for a wave length of 100 km. 

After determining the source parameters, the use of “smart” numerical calculation technologies (hardware acceleration due to parallel FPGA-based calculations [32]) makes it possible to estimate the distribution of maximum wave heights along the coast in a few minutes. 

For example, in the numerical calculations of the 11 March 2011 tsunami propagation on a 2401 × 2401 computational grid, the results of which are shown in Figure 4 and Figure 6, the calculation of one time step lasted 4.72 milliseconds. The total calculation time of 18,000 steps (9000 s of tsunami propagation) is 84.972 s.

The results of numerical simulation of the tsunami generated by the source from [35], using the FPGA-based approach described here (Figure 6), agree quite well with the results of the similar simulation performed by Adriano et al. [35]. The distribution of maximum tsunami heights presented there (Figure 8), qualitatively and quantitatively, practically coincides with a distribution (presented in Figure 6) obtained using the McCormack difference scheme and FPGA-based code acceleration [31]. In this case, the performance of the algorithm based on the FPGA board for the PC corresponds to the performance of a supercomputer.

The detailed wave heights in fine grid-nodes at the sites of special interest can be obtained using a nested-grids approach. Calculations are performed on one or several personal computers (PCs), and can therefore be executed even in cases of power supply failure due to the use of uninterrupted power supplies. To obtain the distribution of the maximum wave heights over a large length of the coastline, it is simply necessary to carry out the computations locally on the required number of PCs equipped with specialized FPGA-based calculators.

## 5. Conclusions

Thus, using data from deep-ocean based sea level detectors makes it possible to restore approximately the tsunami source parameters. Application of a series of “smart” optimizations proposed in the paper (namely: positioning of the observing system and determination of source parameters taking into account a part of the first wave period) can shorten the time required for this stage of the tsunami hazard assessment.

The next stage includes numerical wave propagation modeling (using the obtained source) to estimate the expected tsunami height distribution along the coast of the warning system’s area of responsibility. The developed hardware–software tool on a base of FPGA, which was tested on exact solutions and other methods, makes it possible to reduce computation time down to 1–2 min. Calculation of wave propagation on a PC with hardware acceleration will allow us to approach the problem of timely warning of the population (and industries) about the tsunami wave danger, even in the case of near-zone events. Use of the proposed approach is limited by the size of computation grid compared to eight million nodes. 

## Figures and Tables

**Table 1:** Time (in seconds), necessary to determine source parameters of *CSi* using optimal sensors choice for the cases of one and two working sensors (yellow arrows in Figure 2).

	CS1	CS2	CS3
L5	394	354	478
L3 + L10	296	262	317
L1 + L3 + L10	231	262	317

**Table 2:** Time (in seconds), in which the wave approaches the nearest coast after source parameters are determined for *CSi* sources using optimal sensors for the cases of one and two sensors (red arrows in Figure 2).

	CS1	CS2	CS3
L2	477	528	466
L1 + L4	488	567	708
L1 + L3 + L10	488	624	811

## Data Availability

Not applicable.
